# Supplementary material for: Correction of Significant Urethral Anomalies Using a Tissue-Engineered Human Urethral Substitute: Proof of Concept
Source: Int J Mol Sci. 2025 Feb 20;26(5):1825. doi: 10.3390/ijms26051825 (PMC11899107; doi:10.3390/ijms26051825)
Supplement: Supplementary file 1 [file ijms-26-01825-s001.zip › ijms-3380011-supplementary.pdf]

Correction of Significant Urethral Anomalies Using a Tissue-Engineered Human Urethral Substitute: Proof of Concept (Christophe Caneparo et al. IJMS 2025)

S1. Material and Methods:

The treatment of rabbits with 1 mg/kg of Tacrolimus (FK506, Sigma–Aldrich, Mississauga, ON, Canada) was initiated one week prior to surgeries and administered until the end of the protocol. To determine the efficacy of the immunosuppression, blood samples were collected before oral administration, in EDTA-containing Vacutainer tubes every week following the first surgery for the first month. Then, blood samples were collected once a month for the remainder of the protocol. Samples were immediately frozen until assayed.

The blood level of Tacrolimus was measured using a chromatographic system consisting of a UFLC Prominence (Shimadzu Scientific Instruments Inc., Columbia, MD, USA) cou-pled to an API4000 mass spectrometer (AB Sciex, Concord, On, Canada) as previously described [36]. Briefly, 250 µl of water, 100 µl of blood, 250 µl of 0.1 M zinc sulfate, and 500 µl of standard internal solutions were added to a test tube and thoroughly mixed by vortex-ing for 30 sec. The mixture was then incubated for 10 min at room temperature and centrifuged at ~2400 × g for 10 min. The clear supernatant was collected, and 2 ml of 0.1 M HCl was added before solid-phase extraction. The chromatographic separation was achieved with a Luna C8 (Phenomenex, Torrance, CA, USA). The mobile phase consisted of water with 0.1% acetic acid and 10 mM ammonium acetate (solvent A), methanol with 0.1% acetic acid, and 10 mM ammonium acetate (solvent B). The flow rate was set at 0.9 mL/min. The analytes were eluted using the following program: 0–0.5 min, linear gradient 50–97% B; 0.5–2.5 min, isocratic 97% B; 2.5–2.6 min, linear gradient 97–50% B; 2.6–5.5 min, 50% B. For cost-related reasons, the blood levels of tacrolimus were closely assayed only during the pilot study.

S2. Dosage of Blood Tacrolimus

Oral treatment with tacrolimus 1 mg/kg once a day maintained the immunosuppression of rabbits for the entire in vivo study. Blood levels of tacrolimus was monitored during the whole experiment and for the six rabbits of the pilot study (Figure S1). The values globally ranged between 5 to 10 ng/ml, except for the rabbit BO2 with one value above 10 ng/ml and BO5 with four values under 5 ng/ml.

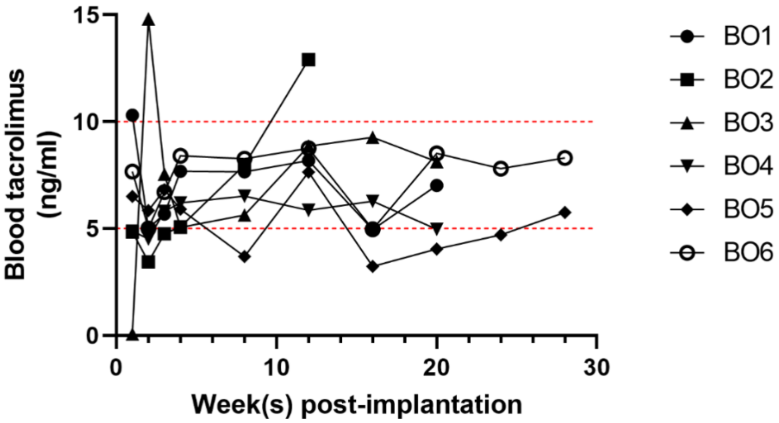

Supplementary Figure S1: Tacrolimus blood levels were measured in immunosuppressed rabbits during the pilot study. The values ranging from 5 to 10 ng/ml of tacrolimus correspond to the optimal immunosuppressive dose (therapeutic window) according to the literature. Rabbits are identified by BO#.
